# Healthcare utilization patterns of individuals with depression after national policy to increase the mental health workforce in primary care: a data linkage study

**DOI:** 10.1186/s12875-024-02402-8

**Published:** 2024-05-08

**Authors:** Jesper T. Dros, Christel E. van Dijk, Koen B.E. Böcker, Lotte C.J.A.F Bruins Slot, Robert A. Verheij, Bert R. Meijboom, Jan-Willem Dik, Isabelle Bos

**Affiliations:** 1 Netherlands Institute for Health Services Research (Nivel), Utrecht, the Netherlands; 2 National Health Care Institute, Diemen, the Netherlands; 3 Tilburg School of Social and Behavioural Sciences, Tilburg University, Tilburg, the Netherlands

**Keywords:** Substitution of Care, Mental Health Nurse, Mental Healthcare, Routine Healthcare Data, Electronic Health Records, Medical Claims Data, Health System reforms, Health Policy

## Abstract

**Background:**

The deployment of the mental health nurse, an additional healthcare provider for individuals in need of mental healthcare in Dutch general practices, was expected to substitute treatments from general practitioners and providers in basic and specialized mental healthcare (psychologists, psychotherapists, psychiatrists, etc.). The goal of this study was to investigate the extent to which the degree of mental health nurse deployment in general practices is associated with healthcare utilization patterns of individuals with depression.

**Methods:**

We combined national health insurers’ claims data with electronic health records from general practices. Healthcare utilization patterns of individuals with depression between 2014 and 2019 (*N* = 31,873) were analysed. The changes in the proportion of individuals treated after depression onset were assessed in association with the degree of mental health nurse deployment in general practices.

**Results:**

The proportion of individuals with depression treated by the GP, in basic and specialized mental healthcare was lower in individuals in practices with high mental health nurse deployment. While the association between mental health nurse deployment and consultation in basic mental healthcare was smaller for individuals who depleted their deductibles, the association was still significant. Treatment volume of general practitioners was also lower in practices with higher levels of mental health nurse deployment.

**Conclusion:**

Individuals receiving care at a general practice with a higher degree of mental health nurse deployment have lower odds of being treated by mental healthcare providers in other healthcare settings. More research is needed to evaluate to what extent substitution of care from specialized mental healthcare towards general practices might be associated with waiting times for specialized mental healthcare.

**Supplementary Information:**

The online version contains supplementary material available at 10.1186/s12875-024-02402-8.

## Introduction

Several diseases currently treated in specialized care could partially be treated in primary care [1–4]. This is also the case in the mental healthcare sector. Policy makers expect that substituting specialized mental health services with primary care is more cost-effective and better for patients in terms of continuity of care as well as travel costs and waiting times [5]. According to the WHO, integration of mental health care in primary care services is important to ensure accessible, affordable and acceptable services to people with mental health problems and their families [6]. They also state that it is an important part of integrated services for mental health to shift care from institutions to ambulatory primary care settings [6]. Substitution of care is defined as “the replacement of (a part of) an existing facility for (a part of) a different facility for the same patient population, while the original function of the facility is maintained” [7]. To be able to substitute specialized care with primary care, capacity and expertise in primary care must be sufficient. However, in many countries, the supply of general practitioners lags behind the growth in demand [8–12]. Increasing the medical workforce in primary care is therefore an important factor for substitution of care to succeed.

In the Netherlands, mental health nurses were introduced in general practice in 2008 to expand the mental health workforce in primary care [5]. Initially limited in number, their deployment rose following an expansion of the capitation fee allowance for general practitioners to hire them in 2014 The policy theory behind this was that an increased mental health workforce in primary care would facilitate substitution, shifting care from basic mental healthcare (BMH) to primary care [5]. Also, national guidelines were formulated stating that individuals in need of mental healthcare could be referred to basic and specialized mental healthcare only with a suspected psychiatric disorder [13]. Individuals without a suspected psychiatric disorder were to be treated in primary care^1^. Mental health nurses are since then tasked with clarifying the symptoms of individuals in need of mental healthcare and deciding whether treatment in general practice or elsewhere in mental healthcare is more appropriate [5]. In Table 1, the role of mental health nurses within the mental healthcare system is depicted.

**Table 1:** Treatment allocation among healthcare providers by patient type in the mental healthcare sector

Healthcare setting	Healthcare provider	Type of mental health problems
General practice	General practitioner	Individuals without psychiatric disorders, with noncomplex psychosocial problems, low risk of (self) harm, and first assessment
	Mental health nurse	Individuals without psychiatric disorder, with somewhat more (complex) psychosocial problems
Basic mental healthcare	(clinical) Psychologists, psychotherapists, nurse specialists, sociopsychiatric nurses	Individuals with a (suspected) psychiatric disorder, medium complexity
Specialized mental healthcare	As BMH, supplemented with: psychiatrists, geriatric physicians, physicians in addiction medicine, peer support, welfare workers, activity supervisors	Individuals with a high risk of (self) harm, highly complex problems, or recurrent symptoms

This table was adapted from Magnée et al. (2017) [5]. Individuals can be treated by several healthcare providers simultaneously

It is important to note that mental health nurses have a wide range of educational backgrounds, which partially overlaps with existing healthcare providers in basic mental healthcare (e.g., sociopsychiatric nurses, psychologists, social workers, nurse specialists) [14]. Therefore, substitution of care is mainly anticipated between healthcare settings (i.e., facilities), not between providers. As treatments by GPs and mental health nurses are exempted from deductible payments (385 euro, annually) in the Netherlands, individuals are incentivized to receive treatment in primary care as opposed to basic or specialized mental healthcare (to which deductibles do apply). The treatments given by mental health nurses encompass for example psychoeducation, lifestyle advice, self-management advice, activity planning. Magnée et al. (2017) argued that mental health nurses have the potential to facilitate substitution of mental healthcare in the Netherlands [15]. They found that a significant number of individuals were treated in the basic or specialized mental healthcare sector without being diagnosed with a psychiatric disorder. In accordance with Table 1, they concluded that this signals a potential to shift these individuals to primary care.

In earlier studies regarding substitution, researcher found that an increase in supply often led to an increase in demand [16]. This is also referred to as Roemers’ Law – an hospital bed built is an hospital bed filled [17, 18]. In other words, initiatives to substitute healthcare services often actually led to complementary healthcare services on top of existing healthcare services. Other studies did find substitution effects between primary care and more specialist care, however [19–21]. The extent to which mental health nurses have actually facilitated substitution of care within mental healthcare has been studied to a limited extent. Previous studies have mainly investigated potential shifts in mental healthcare for the total Dutch population. One study found a 19% decrease in new individuals in specialized mental healthcare between 2012 and 2016 in the Netherlands [22, 23]. At the same time, the number of new individuals in basic mental healthcare remained relatively stable. Between 2015 and 2020, varying numbers were published regarding healthcare use in basic and specialized mental healthcare. One study reported only a slight increase of 2%[24], while another study reported an increase of 10%[25]. After a relatively slow start between 2008 and 2012, the number of new individuals visiting a mental health nurse almost quadrupled between 2012 and 2016[22]. The number of individuals visiting a mental health nurse continued to increase between 2015 and 2020, although less explosively (+ 31%)[24]. Within these studies, the decrease in individuals treated in specialized care is partly attributed to substitution of care. The extent to which these findings are actually related remains uncertain.

In order to gain an in-depth perception of substitution within the mental healthcare sector, specific patient groups need to be examined as opposed to the entire mental healthcare sector. Also, the studies above did not include general practice care (i.e., diagnostic codes, consultations, etc.). In the Netherlands, every citizen is registered at a general practitioner who functions as a gatekeeper. Access to more specialized care is only granted after referral by a general practitioner. This means that GPs are usually the first point of contact within the healthcare system for any given health problem. Individuals can therefore be followed from the start of their treatment journey at their GP through the rest of the healthcare system. Previous studies were limited to series of cross-sectional, monodisciplinary ‘slices’ of these episodes of care. Thus, it remains unclear whether higher mental health nurse deployment was actually associated with a decrease in treatment elsewhere. To evaluate the substitution of care and the role of mental health nurses within this process, healthcare utilization patterns of individuals need to be investigated. Information from the start of the episode of care at the general practitioner, together with diagnostic information, is crucial for the completeness of such analyses.

Therefore, our aim is to study the association between the degree of mental health nurse deployment in general practices and the healthcare utilization patterns of individuals with mental health problems. Our first hypothesis is that individuals in general practices with a high degree of mental health nurse deployment would be treated less often by general practitioners compared to practices with a low degree of mental health nurse deployment. Our second hypothesis is that individuals in general practice with a high degree of mental health nurse deployment would consult basic mental healthcare less often compared to individuals in practices with a low degree of mental health nurse deployment. Opposed to earlier research we were able to include individual patient characteristics instead of analysing results on an aggregated level. Our study will focus on individuals with depression, since depression is among the most common mental health problems, and this is a large patient group that is expected to have a high potential for substitution [26]. As Meeuwissen showed [27], individuals with depression are treated throughout the entire mental health sector through the stepped care principle. This makes this patient group well-suited for care to be shifted between settings within the mental healthcare sector.

## Method

### Study design

We conducted an observational study based on claims data from Dutch health insurers linked with electronic health record (EHR) data from general practitioners (GPs) at the patient level between 2014 and 2019. The EHR data were obtained from GP practices affiliated with Nivel Primary Care Database (Nivel-PCD). Nivel-PCD comprises electronic health records of approximately 10% of the Dutch general practices encompassing an equivalent percentage of approximately 10% of the population. Claims data were provided to the National Health Care Institute (NHCI) by the center for information of Dutch health insurers, Vektis. All medical claims data (both primary and secondary care) of the Dutch insured population under the Health Insurance Act and the Long-Term Care Act are included in claims data at the NHCI. As basic health insurance is mandatory within the Netherlands, these data have nationwide coverage. Data were linked at the patient level with the use of deidentified national citizen service numbers.

### Population

Individuals registered with depression or depressive symptoms (from now on referred to in the paper as being diagnosed with depression^2^) were selected from both the electronic health records and the medical claims data:

1. Individuals treated in general practice were flagged with depression on the basis of the presence of an ICPC [28] code for depression (P76) or depressive symptoms (P03) in their electronic health records;
2. Individuals treated in specialized mental healthcare were flagged in medical claims data of the NHCI, based on the Diagnosis Related Groups (DRGs) [29] for depression in specialized mental healthcare (diagnostic code 011).

Individuals could have multiple episodes of depression within the study period. Any first contact after twelve months without contact for depression was labeled the start of a new or recurrent episode.

However, claims data of basic mental healthcare do not include diagnostic information. Therefore, these data could not be used to select individuals. Nevertheless, we did use them as a starting point of an episode. If the individuals flagged in points 1 and 2 above had a contact in basic mental healthcare no longer than twelve months before their first contact in general practice or specialized mental healthcare, we assumed that this contact was related to depression. For these individuals, the start of an episode was determined by the first contact in basic mental healthcare, provided that individuals had no recordings of other mental health ICPC or DRG codes in the twelve months before this first contact. A schematic overview of this process can be found in Additional file I. Each individual was followed for six months after the onset of an episode. This timespan was chosen to obtain an equal period of follow-up time for each episode. This period represents the initial treatment phase and was chosen based on exploratory data analyses (see Additional file II) and expert opinion of consulted psychologists, psychiatrists and GPs. For all healthcare providers, most individuals are treated within the first couple of months, after which the probability of being treated only increases moderately. Hence, we chose a six-month follow-up so we could analyse individuals with an episode starting up to 2019. We included individuals up until October 2019. In this way, the first wave of COVID-19 in the Netherlands in March 2020 was not part of our study period.

Individuals were included if, in the year before, during and after the initial registration, (I) their age and gender were known and they were 18 years or older, (II) they were registered at a general practice within the Nivel-PCD for the entire year, (III) this practice had provided complete EHR registration data, (IV) this was the only GP practice where they were registered (switching GPs can cause incomplete data), (V) they were not treated in a nursing home (once individuals are treated in a nursing home, they are no longer under formal supervision of their GP) and (VI) their health insurer provided a complete set of claims data for GP care and basic and specialized mental healthcare. A more detailed, schematic overview of this selection process can be found in Additional file III. Individuals with only one contact with their GP and no contact at any other healthcare provider were excluded from the denominator, as these individuals were considered as not being treated for depression.

### Variables

Dependent variables were treatment for depression by a GP, in basic or in specialized mental healthcare (one dichotomous variable per provider). Treatment for depression (recorded with ICPC code P03 or P76) by GPs or mental health nurses was derived from EHRs. First consultations at the general practitioner for an episode of depression were assumed to be ‘gatekeeping’ and were therefore not included as treatment. In this way, individuals who were only seen once by a general practitioner and by no other healthcare provider were not included in the analyses (see Additional file III). Treatment in specialized mental healthcare (registered with diagnostic code 011) was derived from claims data using the DRG classification system for medical claims data [2]. DRGs that were solely diagnostic were excluded from these analyses since these DRGs indicate that no treatment has taken place. Treatment in basic mental healthcare was also derived from claims data. However, since diagnostic information is not registered in basic mental healthcare, all treatments for individuals in our study population were included, thus assuming these treatments were related to the patient’s depression.

Independent variables were age, gender, neighborhood socioeconomic status (SES), depletion of deductibles, psychiatric comorbidities, drug prescriptions and the degree of mental health nurse deployment. These independent variables were included as confounders in our analyses. The latter was defined at the practice level as the number of consultations for individuals with depression recorded by mental health nurses per practice divided by the total number of individuals with depression registered in that practice in that year. No literature is known regarding the level of mental health nurse deployment in general practices. Therefore, quartiles of this deployment were calculated, enabling us to compare practices with relatively low deployment to practices with relatively high deployment. Deductible usage was calculated per patient at the end of the year by subtracting care that is exempt from deductible payments (GP care, midwifery care, care delivered by district nurses)^3^ from the total annual healthcare costs of each patient. An interaction effect was expected between depletion of deductibles and mental health nurse deployment on the extent of substitution for basic and specialized mental healthcare. Therefore, we included an interaction term for the outcomes of being consulted in either basic or specialized mental healthcare. Morbidity data were derived from patient histories as recorded with ICPC codes in EHRs by GPs. The presence of an ICPC code for psychological symptoms (P01-P29) and psychological comorbidities (P70-P99) in the year before or within six months after the start of the depression episode was included in the dataset as a numeric variable (the number of comorbidities within this period). Prescriptions were derived from EHRs of GPs and based on ATC codes [30]. Prescriptions for antidepressants and benzodiazepine were included as individual dichotomous variables (one dichotomous variable per type of prescription). Both comorbidities and prescriptions were included in the analyses to account for differences in complexity^4^. Age, gender and ZIP code were available from the claims data. The neighborhood SES score was also included as relative status scores between neighborhoods divided into quartiles. These scores were derived from education, income and position on the labor market of Dutch inhabitants [3]. The neighborhood’s most recent SES scores were used, which date from 2016.

### Statistical analysis

To investigate development in healthcare utilization at either healthcare provider in primary care or basic or specialized mental healthcare in the period 2014–2019, logistic multilevel regression analyses were conducted with a random intercept at the general practice level (accounting for clustering for repeated measures within general practices) using the lme4 package in R. The main determinant was the degree of deployment of mental health nurses in GP practices at the practice level. All analyses were corrected for gender, age, neighborhood SES, prescriptions and psychological comorbidities. Healthcare utilization patterns in basic and specialized mental healthcare were also corrected for depletion of deductibles, as deductibles were expected to (partially) explain the association between MHN deployment and treatment in basic and specialized mental healthcare. Additionally, the number of treatments per patient with GPs and mental health nurses were calculated. Subgroup analyses were performed for practices with respect to the degree of mental health nurse deployment, which were operationalized in quantiles. Data preparation and linkage were conducted in SAS Enterprise Guide version 7.15; all other analyses were conducted in RStudio version 2021.09.2.

## Results

### Patient characteristics

Most individuals were aged between 18 and 45 years old (47.6%) and predominantly female (62.7%; Table 2 – per year in Additional file IV, Table 1). Compared to the national average, individuals with depression appear to live in neighborhoods with low socioeconomic status more often (30%). On average, 79.5% of individuals with depression had depleted their deductibles. Depletion of deductibles was higher in practices with a lower degree of mental health nurse deployment.

**Table 2:** Patient characteristics (*N* = 31.873), divided into quartiles by degree of mental health nurse deployment between 2014 and 2019

	Mental health nurse deployment							
	Lowest (*N* = 7958)	Low (*N* = 8031)		High (*N* = 7839)		Highest (*N* = 8045)		Overall (*N* = 31,873)
**Female (N, %)**	4947 (62.2)	5086 (63.3)		4857 (62.0)		5089 (63.3)		19,979 (62.7)
**Age (N, %)**								
18–45	3708 (46.6)	3906 (48.6)		3752 (47.9)		3813 (47.4)		15,179 (47.6)
46–64	2927 (36.8)	2888 (36.0)		2726 (34.8)		2910 (36.2)		11,451 (35.9)
65–74	711 (8.9)	690 (8.6)		741 (9.5)		749 (9.3)		2891 (9.1)
75–84	455 (5.7)	407 (5.1)		466 (5.9)		430 (5.3)		1758 (5.5)
85+	157 (2.0)	140 (1.7)		154 (2.0)		143 (1.8)		594 (1.9)
**SES quartiles (N, %)**								
Low	2269 (28.6)	2247 (28.1)		2467 (31.6)		2523 (31.5)		9506 (30.0)
Medium-low	2076 (26.2)	2263 (28.3)		1824 (23.4)		1967 (24.6)		8130 (25.6)
Medium-high	2120 (26.8)	1791 (22.4)		1955 (25.0)		2069 (25.8)		7935 (25.0)
High	1455 (18.4)	1699 (21.2)		1564 (20.0)		1449 (18.1)		6167 (19.4)
**Deductibles depleted (N, %)**	6612 (83.1)	6387 (79.5)		6142 (78.4)		6200 (77.1)		25,341 (79.5)

SES = socioeconomic status

### Healthcare utilization

Large variation in the degree of deployment of mental health nurses is present between practices (Table 3). This variation was relatively stable over time during the study period. Within the first six months after initial contact, most individuals with depression were treated by their GP (Table 3 – per year in Additional file IV, Table 2). While the difference in individuals treated by mental health nurses differs between the quartile with the lowest degree of mental health nurse deployment and the highest degree of mental health nurse deployment (40.6%-point), there is not much difference in individuals treated by the general practitioner (-2.8%-point). Most individuals are treated by both mental health nurses and GPs (Table 3). A larger shift from GPs to mental health nurses is visible in terms of treatment volume. The treatment volume of general practitioners is lower in practices with higher levels of mental health nurse deployment. General practitioners provide 2.70 consultations per individual within six months after diagnosis for practices with the lowest degree of mental health nurse deployment, compared to 2.29 in practices with the highest degree of mental health nurse deployment.

**Table 3:** Treatment of individuals with depression per healthcare provider by degree of mental health nurse deployment in 2014–2019

Mental health nurse deployment					
	Lowest (*N* = 7958)	Low (*N* = 8031)	High (*N* = 7839)	Highest (*N* = 8045)	∆ (%-point)
**Individuals treated by provider (N, %)**					
GP	6555 (82.4)	6640 (82.7)	6409 (81.8)	6407 (79.6)	-2.8
MHN	801 (10.1)	2665 (33.2)	3180 (40.6)	4080 (50.7)	+ 40.6
BMH	1792 (22.5)	1768 (22.0)	1501 (19.1)	1362 (16.9)	-5.6
SMH	2324 (29.2)	2025 (25.2)	1930 (24.6)	1890 (23.5)	-5.7
**Treatment combinations (N, %)**					
GP	3413 (42.9)	2374 (29.6)	2067 (26.4)	1726 (21.5)	-21.4
MHN	37 (0.5)	228 (2.8)	273 (3.5)	458 (5.7)	+ 5.2
GP & MHN	522 (6.6)	1763 (22.0)	2182 (27.8)	2696 (33.5)	+ 26.9
BMH	52 (0.7)	43 (0.5)	29 (0.4)	32 (0.4)	-0.3
GP & BMH	1466 (18.4)	1178 (14.7)	923 (11.8)	683 (8.5)	-9.9
GP & MHN & BMH	124 (1.6)	362 (4.5)	378 (4.8)	477 (5.9)	+ 4.3
SMH	1259 (15.8)	1010 (12.6)	1010 (12.9)	1005 (12.5)	-3.3
GP & SMH	846 (10.6)	652 (8.1)	545 (7.0)	459 (5.7)	-4.9
GP & MHN & SMH	80 (1.0)	214 (2.7)	230 (2.9)	298 (3.7)	+ 2.7
Other combinations	231 (2.9)	297 (3.7)	267 (3.4)	290 (3.6)	+ 0.7
**Number of treatments per patient (mean, SD)**					
GP	2.70 (2.58)	2.47 (2.38)	2.41 (2.46)	2.29 (2.43)	-0.41
MHN	0.25 (0.95)	1.13 (2.14)	1.52 (2.48)	2.17 (2.97)	+ 1.08
**Number of MHN contacts per patient (mean, SD)**	0.14 (0.15)	0.67 (0.11)	1.01 (0.09)	1.51 (0.29)	+ 1.37*

*difference in number of MHN contacts per patient, not %-point. GP = general practitioner; MHN = mental health nurse; BMH = basic mental healthcare; SMH = specialized mental healthcare. Initial contacts are not included as treatment

We can also see a decrease in both the individuals treated in basic and specialized mental healthcare (respectively − 5.6% and − 5.7%). This is reflected in almost all treatment combinations with basic and specialized mental healthcare, except for those where a mental health nurse is involved.

### Association between degree of mental health nurse deployment and care provider

Table 4 shows that higher mental health nurse deployment was associated with lower odds of consultation with a GP in basic mental healthcare and in specialized mental healthcare as opposed to low mental health nurse deployment. For consultations with a GP, only high mental health nurse deployment compared with low mental health nurse deployment was associated with significantly lower odds of consultation. In basic and specialized mental healthcare, higher levels of mental health nurse deployment were consistently associated with lower odds of consultation, as indicated by progressively diminishing Odds Ratios (ORs).

**Table 4:** Association between MHN deployment and being treated by GPs and basic mental healthcare specialized mental healthcare

	General Practitioner			Basic mental healthcare		Specialized mental healthcare	
	*OR*	[95% CI]		*OR*	[95% CI]	*OR*	[95% CI]
**Intercept**	5.35	[4.86 – 5.90]		0.25	[0.22 – 0.27]	0.42	[0.39 – 0.46]
**Mental health nurse deployment (ref: low)**							
Medium-low	1.04	[0.93 − 1.16]		0.90	[0.81 − 1.00]*	0.76	[0.69 − 0.83]***
Medium-high	0.96	[0.86 − 1.08]		0.77	[0.69 − 0.86]***	0.74	[0.67 − 0.81]***
High	0.87	[0.78 − 0.98]**		0.68	[0.61 − 0.77]***	0.73	[0.66 − 0.81]***
**Deductibles depleted**	0.59	[0.57 − 0.61]***		0.72	[0.68 − 0.76]***	1.16	[1.09 − 1.22]***
**Deductibles depleted** *MHN deployment							
Depleted*Medium-low				1.11	[1.03 − 1.20]**	1.02	[0.95 − 1.10]
Depleted*Medium-high				1.16	[1.08 − 1.25]***	1.06	[0.98 − 1.15]
Depleted*High				1.18	[1.09 − 1.27]***	1.12	[1.04 − 1.21]**

*P < 0.05, **P < 0.01, ***P < 0.001. MHN = mental health nurse. **§** Also corrected for the depletion of deductibles. The odds ratios depicted in the table are the results of the main outcome of the multilevel regression with a random intercept on practice level, corrected for individual age, gender, socioeconomic status, number of prescriptions and comorbidities. Full regression outputs can be found in Additional file V

However, both the effects of mental health nurse deployment on basic and on specialized mental healthcare were modified by depletion of deductibles, as indicated by a significant interaction term in Table 4. For basic mental healthcare, depletion of deductibles reduced the odds of consultation for all level of mental health nurse deployment. The strongest reduction was found for individuals who had depleted their deductibles in practices with the lowest deployment of mental health nurses, with 0.18 odds (0.25*0.72) as compared to 0.25 odds for individuals who had not depleted their deductibles. For specialized mental healthcare, depletion of deductibles increased the odds of consultation – but was only significant with the highest level of mental health nurse deployment. The difference for individuals who had depleted their deductibles in practices with the lowest deployment of mental health nurses, was 0.41 odds (0.43*0.73*1,16*1,12) as compared to 0.31 odds (0.43*0.73) for individuals who had not depleted their deductibles. Calculations of ORs for all combinations of mental health nurse deployment and depletion of deductibles can be found in Additional File V.

## Discussion

We aimed to study the association between the degree of mental health nurse deployment within general practices and the allocation of treatment within the mental health system. Individuals receiving care at a general practice with a higher degree of mental health nurse deployment had lower odds of being treated by mental healthcare providers in more specialized healthcare settings as compared to individuals receiving care at a general practice with a low degree of mental health nurse deployment. In general practices with a higher degree of mental health nurses, individuals with depression were less often treated in basic and specialized mental healthcare and more often by mental health nurses. Within general practices, mental health nurses seemed to alleviate the treatment workload for the general practitioner, but only when mental health nurse deployment was high.

Our first hypothesis was that for individuals receiving care in general practices with a higher degree of mental health nurse deployment, treatments would be provided less by GPs and more by mental health nurses compared to individuals in practices with a low degree of mental health nurse deployment. This hypothesis was confirmed. Additionally, the number of consultations with GPs per patient is lower with higher mental health nurse deployment. However, GPs remain involved in the treatment process in most cases, which is in line with the earlier research [31] and functional profiles and collaboration agreements of mental health nurses and general practitioners [32]. While the treatment workload is alleviated, this does not necessarily imply an overall decrease in GP workload. While the number of consultations decreases, GPs often remains involved and also gets additional management and administrative tasks regarding mental health nurses [31]. This was also found by a qualitative study on healthcare trajectories for individuals with depression [33], and might be problematic considering the high workload present among Dutch GPs [34].

Our second hypothesis was that individuals in practices with high mental health nurse deployment would be treated less often in basic and specialized mental health care. This hypothesis is supported by our findings, as the deployment of mental health nurses seems to facilitate substitution from basic and specialized mental healthcare toward primary care. The progressively diminishing trend of being treated was strongest for basic mental healthcare. This differential response might imply that higher nurse deployment exerts incremental influence on basic mental healthcare utilization, which highlights the importance of optimal mental health nurse deployment in achieving substitution. In contrast, specialized mental healthcare showed a strong association even at medium-low deployment levels, suggesting that high mental health nurse deployment might not be necessary in order to achieve substitution with specialized mental healthcare. While depletion of deductibles diminishes the association between mental health nurse deployment and basic mental healthcare, it had little impact on the association between mental health nurse deployment and specialized mental healthcare (see Additional File V). Hence, more than merely financial incentives to receive healthcare services in general practice – which is exempted from paying deductibles – seem to explain this shift. As there is much overlap between the educational backgrounds of healthcare providers in basic mental healthcare and mental health nurses [14], it is important to note that substitution of care from basic mental healthcare toward mental health nurses is mainly substitution between healthcare settings.

In an earlier study, Magnée et al. (2016) did not find a shift in care from GPs to mental health nurses [35]. However, they used another method to define the degree of mental health nurse deployment. They compared practices with at least one mental health nurse (defined as at least 25 mental health nurse consultations per practice in a given year) to practices without a mental health nurse (defined as less than 25 mental health nurse consultations per practice in a given year). In our study, we have more differentiation within the degree of mental health nurse deployment, which could explain the different outcomes. In another study, Magnée et al. (2017) reported a potential for substitution of mental healthcare with general practices, enabled by the introduction of mental health nurses, among others [15]. Our study indicates that this potential may at least be partly fulfilled for individuals with depression. The association between mental health nurse deployment and basic and specialized mental healthcare, as reported in our study, could also be due to an increase in individuals with depression. However, the Netherlands Mental Health Survey and Incidence Study (NEMESIS) reports a relative increase in the use of primary healthcare services compared to secondary healthcare services that cannot be explained by the prevalence of psychological disorders between 2007 and 2009 and 2019-2022 [25]. To our knowledge, no other studies exist on the association between mental health nurse deployment and specialized healthcare for individuals in need of mental healthcare. A systematic review on the substitution of physicians by nurses in primary care found that nurse-led care was effective in reducing the overall risk of hospital admission, albeit not specifically for mental healthcare [36]. However, a Cochrane Review not specifically focused on mental healthcare found little or no difference in the number of hospital referrals and hospital admissions between nurses and doctors [37].

### Strengths and limitations

The main strength of our study is the use of routinely recorded healthcare data, which enabled us to include a representative large study population and to construct detailed healthcare utilization patterns of individuals who were diagnosed with depression by a healthcare professional (i.e., not self-reported). There were also a few limitations. We used an observational study method, so no causal relationships between mental health nurse deployment and differences in healthcare utilization patterns could be examined. Additionally, not all factors potentially influencing healthcare utilization patterns can be derived from routinely recorded healthcare data. First, we had no information on waiting times for basic and specialized mental healthcare. Longer waiting times could have resulted in more individuals being treated within general practice. Therefore, an alternative hypothesis is that the association between the degree of deployment of mental health and treatment in basic and specialized mental healthcare might be related to the extent to which waiting times are present. However, regional waiting times in basic and specialized mental healthcare are only reported from 2019 onwards and could therefore not be included in this study. Second, we had no information on diagnosis in basic mental healthcare. Therefore, we may have included some basic mental healthcare for other mental health conditions. The share of individuals treated for depression in basic mental healthcare in our study is therefore overestimated. However, the degree of overestimation is unlikely to differ between practices with a high or low degree of mental health nurse deployment. Third, we had little information regarding the complexity of depression. We attempted to account for complexity by taking prescriptions and psychological comorbidities into account. Other relevant factors for the complexity of individuals relative to substitution of care that could not be included are, for example, preferences of the individual, level of health literacy, social network and patient-provider relationship, among others [31]. Lastly, we selected individuals who have received a diagnostic code for depression or depressive symptoms. Presumably, mental health nurses also provide treatment to individuals who are perhaps being assessed for having depression, and thus might not receive diagnoses. Our study underestimates the number of patients for which mental health nurses could provide treatment.

## Conclusion

In general practices with higher deployment of mental health nurses, more individuals with depression are treated by the mental health nurse and less by the GP compared with general practices with lower deployment of mental health nurses. Additionally, these individuals were less often treated within basic and specialized mental healthcare. Future research should focus on the generalizability of our results to other mental health problems.

### Policy implications

Our research might indicate that policies to reinforce the mental health workforce in primary care could be useful to increase primary care relative to basic and specialized mental healthcare. If policy makers desire to stimulate primary care relative to other healthcare domains, considering incentives to increase the workforce in primary care could therefore be beneficial. However, since all healthcare sectors differ, contextual factors like reimbursement types and organization of care should always be taken into account when designing such policy measures.

### Electronic supplementary material

Below is the link to the electronic supplementary material.

Supplementary Material 1

## Data Availability

The data that support the findings of this study are available from Nivel and the NHCI, but restrictions apply to the availability of these data, which were used under license for the current study and so are not publicly available. According to the GDPR, the National Health Care Institute has a legal basis to process this health data. Datasets are only available for organizations with a legal basis to process this health data which is in accordance with the legal basis for which the National Health Care Institute has collected the data. Data at the aggregated level are available from the corresponding author upon request and with the permission of Nivel and the NHCI.

## Footnotes

1 A suspected psychiatric disorder is an important criterium for referral to basic- or specialized mental healthcare, however it is not the only criterium. Criteria are, among others, for the suspected psychiatric disorder to be accompanied by moderate or severe suffering and no improvement after treatment in general practice [13].

2 The authors acknowledge that being registered with depression or depressive symptoms does not always equal an official diagnosis with depression in clinical practice. Additionally, absence of a registration with depression or depressive symptoms does not always mean an official diagnosis with depression is absent. However, after extensive consultation of clinical experts and data experts, this method was found to portray the closets depiction of reality.

3 For the calculation of deductible depletion for the outcome of consultation in basic or specialized mental healthcare, we also subtracted basic or specialized mental healthcare costs, respectively.

4 Comorbidities and prescriptions are not the only factors contributing to complexity. However, other factors like personal and contextual circumstances, cannot be retrieved from routine healthcare data and could therefore not be included in analyses.

## Declarations

Protection Regulation (GDPR) is adhered to. Steering committees with representatives from healthcare providers’ national associations take part in the decision-making process of the use of data (see https://www.Nivel.nl/en/Nivel-zorgregistraties-eerste-lijn/Nivel-primary-care-database for more information). This study was approved according to the governance code of the Nivel Primary Care Database under project number NZR-00321.059. The National Health Care Institute has a legal basis to process these health data, in accordance with the GDPR. Deidentified (claims) data is personal data according to the GDPR, but can’t be traced back to individual citizens. The National Health Care Institute uses the deidentified data for its legal tasks; no objection or individual complaint can be submitted.

## References

[1] Quanjel TCC, Spreeuwenberg MD, Struijs JN, Baan CA, Ruwaard D. Substituting hospital-based outpatient cardiology care: The impact on quality, health and costs. Tu WJ, ed. *PLOS ONE*. 2019;14(5):e0217923. 10.1371/journal.pone.0217923 10.1371/journal.pone.0217923 PMC6544378 31150520

[2] van Hoof (2016). Substitution of Hospital Care with Primary Care: defining the conditions of Primary Care Plus. Int J Integr Care.

[3] Winpenny EM, Miani C, Pitchforth E, King S, Roland M (2017). Improving the effectiveness and efficiency of outpatient services: a scoping review of interventions at the primary–secondary care interface. J Health Serv Res Policy.

[4] Roland PM, McDonald DR, Sibbald PB. A report to the NHS service delivery and Organisation R&D Programme from the National Primary Care Research and Development Centre and Centre for Public Policy and Management of the University of Manchester March 2006. Published online 2006:254.

[5] Magnée T, Verhaak PFM, Rijksuniversiteit, Groningen. Nederlands instituut voor onderzoek van de gezondheidszorg (Utrecht). *Mental Health Care in General Practice in the Context of a System Reform*.; 2017.

[6] World Health Organization. Mental health in primary care: illusion or inclusion? Published 2018. Accessed August 15. 2023. https://www.who.int/docs/default-source/primary-health-care-conference/mental-health.pdf?sfvrsn=8c4621d2_2

[7] van Dijk CE, Korevaar JC, de Jong JD, Koopmans B, van Dijk M, de Bakker D. Kennisvraag Substitutie Tweede Naar Eerste Lijn. Published online 2013.

[8] Kringos D, Boerma W, Bourgueil Y (2013). The strength of primary care in Europe: an international comparative study. Br J Gen Pract.

[9] Kuhlmann E, Groenewegen PP, Bond C, Burau V, Hunter DJ (2018). Primary care workforce development in Europe: an overview of health system responses and stakeholder views. Health Policy.

[10] Majeed A. Shortage of general practitioners in the NHS. *BMJ*. Published online July 10, 2017:j3191. 10.1136/bmj.j3191 10.1136/bmj.j3191 28694250

[11] The Complexities of Physician Supply and Demand. Projections From 2018 to 2033. Published online 2018:92.

[12] Capaciteitsorgaan. *Capaciteitsplan 2021–2024 Deelrapport 2 Huisartsgeneeskunde*.; 2019.

[13] Netwerk Kwaliteitsontwikkeling GGz. Landelijke samenwerkingsafspraken tussen huisarts, generalistische basis GGz en gespecialiseerde GGz. Published 2016. Accessed August 2. 2023. https://www.ggzcentraal.nl/wp-content/uploads/2017/12/lga-opmaak_def.pdf

[14] Nuijen J, Kenter A, Ringoir L. Organisatie en uitvoering van de functie POH-GGZ in de praktijk.

[15] Magnée T, de Beurs DP, Boxem R, de Bakker DH, Verhaak PF (2017). Potential for substitution of mental health care towards family practices: an observational study. BMC Fam Pract.

[16] Van Den Bogaart EH, Spreeuwenberg MD, Kroese ME, Ruwaard D (2023). Substitution or addition: an observational study of a new primary care initiative in the Netherlands. J Health Serv Res Policy.

[17] Delamater PL, Messina JP, Grady SC, WinklerPrins V, Shortridge AM. Do More Hospital Beds Lead to Higher Hospitalization Rates? A Spatial Examination of Roemer’s Law. Fielding R, ed. *PLoS ONE*. 2013;8(2):e54900. 10.1371/journal.pone.0054900 10.1371/journal.pone.0054900 PMC3572098 23418432

[18] Shain R. Hospital costs relate to the supply of beds. Mod Hosp. 1959;(92):71–3. 13644010

[19] Atella V, Deb P (2008). Are primary care physicians, public and private sector specialists substitutes or complements? Evidence from a simultaneous equations model for count data. J Health Econ.

[20] Fortney JC, Steffick DE, Burgess JF, Maciejewski ML, Petersen LA (2005). Are primary Care Services a Substitute or Complement for Specialty and Inpatient services? Are primary Care services a substitute or complement?. Health Serv Res.

[21] Dros JT, Van Dijk CE, Bos I (2023). Healthcare utilization patterns for knee or hip osteoarthritis before and after changes in national health insurance coverage: a data linkage study. Health Policy.

[22] KPMG. Eerste resultaten Substitutiemonitor 2015. Published online 2015.

[23] *Monitor generalistische basis GGZ*. KPMG; 2018.

[24] Ggz uit de knel. Published online 2022.

[25] Ten Have M, Tuithof M, Van Dorsselaer S, Schouten F, Luik AI, De Graaf R (2023). Prevalence and trends of common mental disorders from 2007-2009 to 2019‐2022: results from the Netherlands Mental Health Survey and Incidence studies (NEMESIS), including comparison of prevalence rates before vs. during the COVID‐19 pandemic. World Psychiatry.

[26] Depressie | NHG-Richtlijnen. Accessed March 30. 2023. https://richtlijnen.nhg.org/standaarden/depressie

[27] Meeuwissen JAC. *The Case for Stepped Care: Exploring the Applicability and Cost-Utility of Stepped-Care Strategies in the Management of Depression*. 2018.

[28] NHG. *NHG-Richtlijn Adequate Dossiervorming Met Het Elektronisch Patiëntdossier (ADEPD)*.; 2012.

[29] Hasaart F. Incentives in the diagnosis treatment combination payment system for specialist Medical Care: a study about behavioral responses of medical specialists and hospitals in the Netherlands. Maastricht University; 2011.

[30] WHOCC - ATC/DDD Index. Accessed March 30. 2023. https://www.whocc.no/atc_ddd_index/

[31] Noordam D, Heijmans M, van Dulmen S. Zorgtrajecten depressie en persoonlijkheidsproblematiek vanuit de huisartspraktijk.

[32] LV POH-GGZ. Definitief Functie en competentieprofiel POH GGZ 2020. Accessed August 2. 2023. https://poh-ggz.nl/wp-content/uploads/2020/03/Definitief-Functie-en-competentieprofiel-POH-GGZ-2020-versie-1.0-04032020.pdf

[33] Noordam D, Heijmans M, van Dulmen S. Mental healthcare and shared decision-making in Dutch primary care.

[34] Damen L, van Tuyl L, de Jong JD. General practitioners’ perspectives on relocating care: a Dutch interview study. *Submitted*. 10.1186/s12875-024-02425-1 PMC11127345 38796424

[35] Magnée T, de Beurs DP, de Bakker DH, Verhaak PF (2016). Consultations in general practices with and without mental health nurses: an observational study from 2010 to 2014. BMJ Open.

[36] Martínez-González NA, Djalali S, Tandjung R (2014). Substitution of physicians by nurses in primary care: a systematic review and meta-analysis. BMC Health Serv Res.

[37] Laurant M, van der Biezen M, Wijers N, Watananirun K, Kontopantelis E, van Vught AJ. Nurses as substitutes for doctors in primary care. Cochrane Database Syst Rev. 2018;2019(7). 10.1002/14651858.cd001271.pub3 10.1002/14651858.CD001271.pub3 PMC6367893 30011347

