# Supplementary material for: Healthcare utilization patterns of individuals with depression after national policy to increase the mental health workforce in primary care: a data linkage study
Source: BMC Prim Care. 2024 May 8;25:158. doi: 10.1186/s12875-024-02402-8 (PMC11077842; doi:10.1186/s12875-024-02402-8)

**Additional file I: patient selection and exclusion criteria**

*Start of an episode in basic mental healthcare*

In routine healthcare data from basic mental healthcare, no information regarding the diagnosis of the individuals are provided. However, basic mental healthcare is an essential part of the mental healthcare system. Therefore, we decided to still include basic mental healthcare to determine the start of an episode, in the best way possible:

1. If individuals had had a consultation in basic mental healthcare in the year before the start of a depression episode in general practice or in specialized mental healthcare (see patient 1 and 2 in the table below), we assumed that this consultation was related to depression. In this case, the first BMH contact was considered to be the start of the episode.
2. An exception to the statement above in point 1 was made if, 1) before the consultation in basic mental healthcare or 2) between the consultation in basic mental healthcare and the start of an episode in general practice or specialized mental healthcare, an individual had had an episode for a different psychological complaint than depression. In that case, we assumed that the consultation in basic mental healthcare was not related to depression (see patient 3 below). In this case, the first GP or SMH contact was considered to be the start of the episode.

We have illustrated this below:

**Additional file II: follow-up time**

Each individual was followed for six months after the onset of an episode. This timespan was chosen in order to obtain an equal period of follow-up time for each episode. This period represents the initial treatment phase and was chosen based on expert opinion of consulted psychologists, psychiatrists and GPs, combined with exploratory data analyses that can be seen below.

For individuals with an episode starting in 2014, we made a Kaplan-Meijer Curve for the probability of not being treated at the end of the study period in 2019. We selected only individuals that had complete data over the entire study period. For all healthcare providers, it can be seen that the probability of not being treated decreases the strongest within the first couple of months, after which this probability stabilizes. The x-axis shows the time in days after the start of an episode.


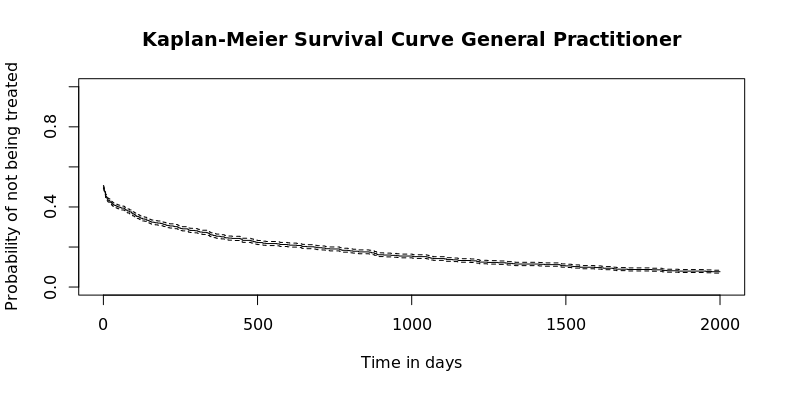


Note that for the general practitioner, most individuals are treated when time in days equals 0. This is expected, since most individuals start their treatment journey at the general practitioner. Also, almost all individuals are treated by the general practitioner at the end of the study period (i.e. within 6 years).


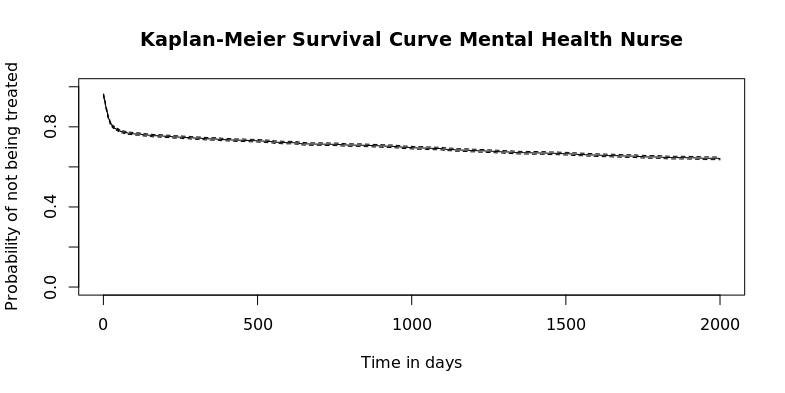


Especially at the mental health nurse, most individuals are seen within the first months.


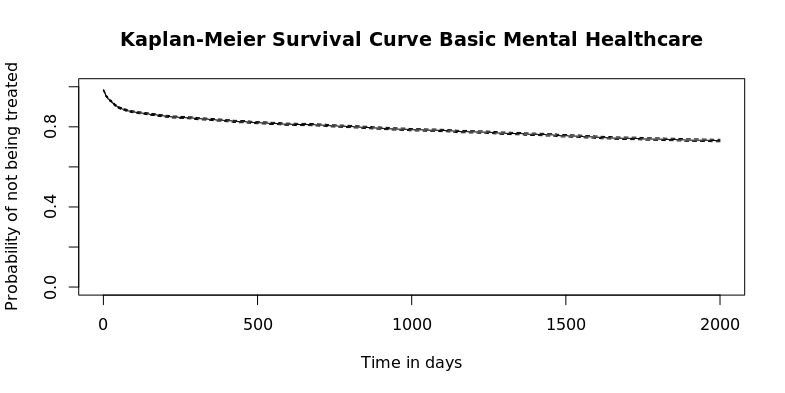


For basic mental healthcare, even though relative stabilization can be seen after some months, this is less distinct than at other healthcare providers. Likely this is due to the fact that this is due to an overestimation of individuals with depression, since diagnostic information in basic mental healthcare is lacking.


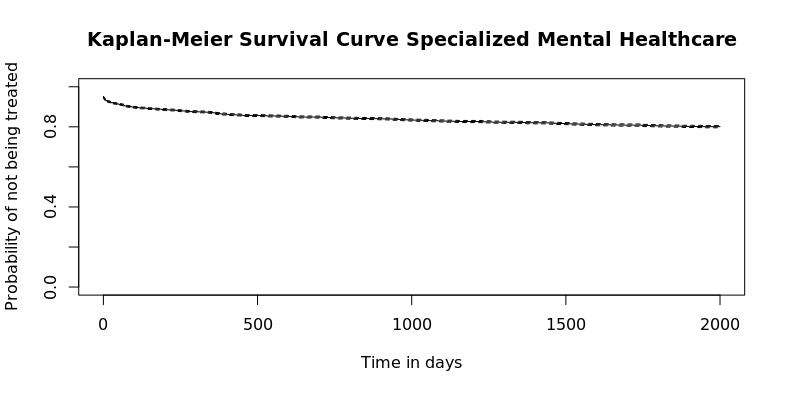


**Additional file III: exclusion per criterium**

| **Year** | **All individuals** | **Depressive episode in basic mental healthcare** | | **Registered in general practice within the Nivel-PCD** | | **Registered at health insurer within medical claims data** | | **Not treated in a nursing home; general practitioner no longer main healthcare provider** | | **No episode during first wave of COVID-19; deviation in healthcare usage** | | **Not insured at Menzis or DSW: these insurers use a capitation model in general practice, for which health services not registered** | | **Age 18 or older** | | **>1 consultation at the GP; only 1 consultation at the GP was assumed to be gatekeeping** | |
| --- | --- | --- | --- | --- | --- | --- | --- | --- | --- | --- | --- | --- | --- | --- | --- | --- | --- |
|  | N | % | N | % | N | % | N | % | N | % | N | % | **N** | % | **N** | % | **N** |
| 2014 | 26938 | 89,5% | 24116 | 45,9% | 11059 | 85,8% | 9485 | 99,3% | 9423 | 100,0% | 9423 | 100,0% | 9423 | 95,1% | 8963 | 69,2% | 6200 |
| 2015 | 23257 | 91,8% | 21357 | 45,5% | 9725 | 87,2% | 8479 | 99,4% | 8428 | 100,0% | 8428 | 99,1% | 8350 | 95,8% | 8001 | 69,8% | 5588 |
| 2016 | 19615 | 90,7% | 17799 | 50,5% | 8992 | 92,1% | 8280 | 99,5% | 8241 | 100,0% | 8241 | 93,2% | 7682 | 94,9% | 7292 | 68,5% | 4996 |
| 2017 | 25289 | 94,0% | 23761 | 35,1% | 8347 | 92,5% | 7717 | 99,7% | 7696 | 100,0% | 7696 | 92,3% | 7106 | 94,2% | 6692 | 69,7% | 4666 |
| 2018 | 22220 | 94,9% | 21085 | 45,1% | 9509 | 88,6% | 8426 | 99,6% | 8395 | 100,0% | 8395 | 96,0% | 8063 | 94,2% | 7598 | 68,0% | 5164 |
| 2019 | 24963 | 98,3% | 24527 | 46,9% | 11499 | 96,5% | 11095 | 99,8% | 11071 | 75,0% | 8302 | 99,2% | 8233 | 94,5% | 7781 | 67,6% | 5259 |

The table above is depicted as a flowchart for 2019 on the following page, for clarification purposes.


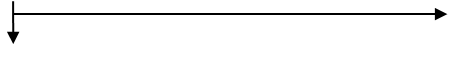

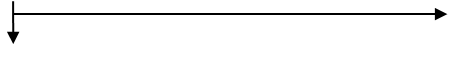

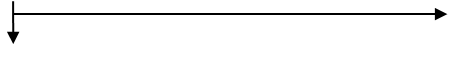

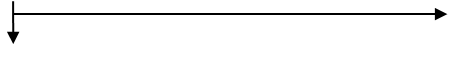

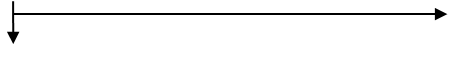

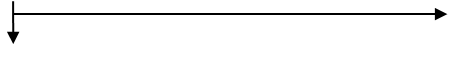


**Not insured at Menzis or DSW; health services not registered (N=69)**

**Not treated during first wave of COVID-19; deviation in healthcare usage (N=2769)**

**No depressive episode in basic mental healthcare (N=436)**

**Not treated in a nursing home; general practitioner no longer main healthcare provider (N=24)**

**Not registered at health insurer within medical claims data (N=404)**

**Not registered in general practice within the Nivel-PCD (N=13028)**

**N=24527**

**N=11499**

**N=11095**

**N=11071**

**N=8302**

**All individuals (2019)**

**N= 24963**


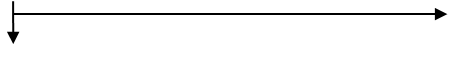

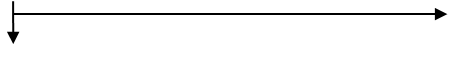


**N=7781**

**Age <18 (N=452)**

**<2 consultation at the GP; only 1 consultation at the GP was assumed to be gatekeeping (N=2522)**

**N=8233**

**N=5259**

**Additional file IV: patient and treatment characteristics per year during study period**

Table 1. patient characteristics.

|  | **2014**  **(N=6200)** | **2015**  **(N=5588)** | **2016**  **(N=4996)** | **2017**  **(N=4666)** | **2018**  **(N=5164)** | **2019**  **(N=5259)** | **Overall**  **(N=31873)** |
| --- | --- | --- | --- | --- | --- | --- | --- |
| **Female (N, %)** | 3916 (63.2) | 3524 (63.1) | 3098 (62.0) | 2918 (62.5) | 3255 (63.0) | 3268 (62.1) | 19979 (62.7) |
| **Age (N, %)** |  |  |  |  |  |  |  |
| 18-45 | 3057 (49.3) | 2576 (46.1) | 2372 (47.5) | 2119 (45.4) | 2488 (48.2) | 2567 (48.8) | 15179 (47.6) |
| 46-64 | 2244 (36.2) | 2049 (36.7) | 1831 (36.6) | 1717 (36.8) | 1836 (35.6) | 1774 (33.7) | 11451 (35.9) |
| 65-74 | 502 (8.1) | 495 (8.9) | 463 (9.3) | 475 (10.2) | 459 (8.9) | 497 (9.5) | 2891 (9.1) |
| 75-84 | 281 (4.5) | 362 (6.5) | 246 (4.9) | 265 (5.7) | 289 (5.6) | 315 (6.0) | 1758 (5.5) |
| 85+ | 116 (1.9) | 106 (1.9) | 84 (1.7) | 90 (1.9) | 92 (1.8) | 106 (2.0) | 594 (1.9) |
| **SES quartiles (N, %)** |  |  |  |  |  |  |  |
| Low | 1866 (30.2) | 1755 (31.6) | 1447 (29.1) | 1295 (27.9) | 1498 (29.1) | 1645 (31.5) | 9506 (30.0) |
| Medium-low | 1709 (27.7) | 1560 (28.1) | 1277 (25.7) | 1143 (24.6) | 1212 (23.5) | 1229 (23.5) | 8130 (25.6) |
| Medium-high | 1427 (23.1) | 1427 (25.7) | 1426 (28.7) | 1210 (26.0) | 1238 (24.0) | 1207 (23.1) | 7935 (25.0) |
| High | 1173 (19.0) | 817 (14.7) | 825 (16.6) | 999 (21.5) | 1204 (23.4) | 1149 (22.0) | 6167 (19.4) |
|  |  |  |  |  |  |  |  |
| **Deductibles depleted (N, %)** | 4898 (79) | 4414 (79) | 3897 (78) | 3732 (80) | 4079 (79) | 4312 (82) | 25498 (80) |

**SES= socioeconomic status.**

Table 2. Treatment of individuals with depression within six months after first contact per healthcare provider per year. Initial contacts are not included as treatment.

|  | **2014**  **(N=6200)** | **2015**  **(N=5588)** | **2016**  **(N=4996)** | **2017**  **(N=4666)** | **2018**  **(N=5164)** | **2019**  **(N=5259)** | **∆**  **(%-point)** |
| --- | --- | --- | --- | --- | --- | --- | --- |
| **Treatment <6 months after initial contact N (%)** |  |  |  |  |  |  |  |
| GP | 1958 (31.6) | 1703 (30.5) | 1505 (30.1) | 1294 (27.7) | 1539 (29.8) | 1581 (30.1) | -1.5 |
| MHN | 170 (2.7) | 170 (3.0) | 117 (2.3) | 190 (4.1) | 163 (3.2) | 186 (3.5) | +0.8 |
| GP & MHN | 1323 (21.3) | 1309 (23.4) | 1041 (20.8) | 1094 (23.4) | 1158 (22.4) | 1238 (23.5) | +2.2 |
| BMH | 53 (0.9) | 21 (0.4) | 31 (0.6) | 22 (0.5) | 27 (0.5) | 2 (0.0) | -0.9 |
| GP & BMH | 897 (14.5) | 741 (13.3) | 752 (15.1) | 572 (12.3) | 673 (13.0) | 615 (11.7) | -2.8 |
| SMH (outpatient) | 812 (13.1) | 663 (11.9) | 672 (13.5) | 700 (15.0) | 675 (13.1) | 735 (14.0) | +0.9 |
| GP + SMH (outpatient) | 490 (7.9) | 387 (6.9) | 404 (8.1) | 297 (6.4) | 372 (7.2) | 338 (6.4) | -1.5 |
| Other combinations | 4960 (8.0) | 5923 (10.6) | 4746 (9.5) | 4946 (10.6) | 5577 (10.8) | 5679 (10.8) | +2.0 |
| **Treatments combined N (%)** |  |  |  |  |  |  |  |
| GP | 5101 (82.3) | 4654 (83.3) | 4113 (82.3) | 3681 (78.9) | 4224 (81.8) | 4238 (80.6) | -1.7 |
| MHN | 1876 (30.3) | 1955 (35.0) | 1534 (30.7) | 1694 (36.3) | 1773 (34.3) | 1894 (36.0) | +5.7 |
| BMH | 1259 (20.3) | 1141 (20.4) | 1081 (21.6) | 898 (19.2) | 1062 (20.6) | 982 (18.7) | -1.6 |
| SMH (outpatient) | 1518 (24.5) | 1289 (23.1) | 1286 (25.7) | 1222 (26.2) | 1278 (24.7) | 1304 (24.8) | +0.3 |
| SMH (inpatient) | 66 (1.1) | 80 (1.4) | 62 (1.2) | 41 (0.9) | 58 (1.1) | 54 (1.0) | -0.1 |
| **Number of MHN contacts per patient (mean, SD)** | 0.76 (0.52) | 0.84 (0.53) | 0.70 (0.56) | 0.90 (0.51) | 0.89 (0.53) | 0.93 (0.50) | 0.17* |

**Additional file V: full regression output**

|  | **General Practitioner** | | | | **Basic mental healthcare** | | | | | **Specialized mental healthcare** | | | | |
| --- | --- | --- | --- | --- | --- | --- | --- | --- | --- | --- | --- | --- | --- | --- |
|  | *OR* | | [95% CI] | | *OR* | | | [95% CI] | | *OR* | | | [95% CI] | |
| Intercept | 5.35 | | [4.86 – 5.90] | | 0.25 | | | [0.22 – 0.27] | | 0.42 | | | [0.39 – 0.46] | |
| **Mental health nurse deployment** |  |  | |  | |  |  | |  | |  |  | |  |
| Medium-low | 1,04 | [0,93 | | 1,16] | | 0,90 | [0,81 | | 1,00]* | | 0,76 | [0,69 | | 0,83]*** |
| Medium-high | 0,96 | [0,86 | | 1,08] | | 0,77 | [0,69 | | 0,86]*** | | 0,74 | [0,67 | | 0,81]*** |
| High | 0,87 | [0,78 | | 0,98]** | | 0,68 | [0,61 | | 0,77]*** | | 0,73 | [0,66 | | 0,81]*** |
| **Age in years** | 1,20 | [1,17 | | 1,24]*** | | 0,74 | [0,72 | | 0,77]*** | | 0,73 | [0,71 | | 0,76]*** |
| **Female (yes/no)** | 1,02 | [0,96 | | 1,09] | | 1,02 | [0,96 | | 1,09] | | 0,87 | [0,83 | | 0,92]*** |
| **Socioeconomic status score** | 1,00 | [0,96 | | 1,03] | | 1,04 | [1,01 | | 1,08]* | | 1,01 | [0,98 | | 1,05] |
| **Deductibles depleted** | 0,59 | [0,57 | | 0,61]*** | | 0,72 | [0,68 | | 0,76]*** | | 1,16 | [1,09 | | 1,22]*** |
|  |  |  | |  | |  |  | |  | |  |  | |  |
| **Deductibles depleted**  ***MHN deployment** |  |  | |  | |  |  | |  | |  |  | |  |
| Depleted*Medium-low |  |  | |  | | 1,11 | [1,03 | | 1,20]** | | 1,02 | [0,95 | | 1,10] |
| Depleted*Medium-high |  |  | |  | | 1,16 | [1,08 | | 1,25]*** | | 1,06 | [0,98 | | 1,15] |
| Depleted*High |  |  | |  | | 1,18 | [1,09 | | 1,27]*** | | 1,12 | [1,04 | | 1,21]** |
| **Number of psychological complaints** | 0,82 | [0,80 | | 0,85]*** | | 0,93 | [0,90 | | 0,97]*** | | 1,13 | [1,10 | | 1,16]*** |
| **Number of psychological disorders** | 0,73 | [0,71 | | 0,75]*** | | 0,97 | [0,94 | | 1,00]* | | 1,26 | [1,23 | | 1,30]*** |
| **Benzodiazepine (yes/no)** | 1,11 | [1,07 | | 1,15]*** | | 1,03 | [1,00 | | 1,07]* | | 1,14 | [1,11 | | 1,18]*** |
| **Antidepressant (yes/no)** | 1,25 | [1,21 | | 1,29]*** | | 0,78 | [0,76 | | 0,81]*** | | 1,22 | [1,19 | | 1,26]*** |

Table 1. Full regression output for being treated at the general practitioner, in basic mental healthcare of in specialized mental healthcare as a function of mental health nurse deployment, corrected for age, gender, socioeconomic status, depletion of deductibles (including interaction term), psychological comorbidities and medication usage.

*P<0.05, **P<0.01, ***P<0.001

|  | **Basic mental healthcare** | | | **Specialized mental healthcare** | | | |
| --- | --- | --- | --- | --- | --- | --- | --- |
|  | *OR* | [95% CI] | | *OR* | | [95% CI] | |
| Intercept | 0.25 | [0.22 – 0.27] | | 0.43 | | [0.39 – 0.47] | |
| **Mental health nurse deployment** |  |  |  |  |  | |  |
| Medium-low | 0,91 | [0,82 | 1,01] | 0,75 | [0,69 | | 0,83]*** |
| Medium-high | 0,79 | [0,71 | 0,88]*** | 0,73 | [0,66 | | 0,81]*** |
| High | 0,70 | [0,63 | 0,79]*** | 0,73 | [0,65 | | 0,80]*** |
|  |  |  |  |  |  | |  |
| **Deductibles depleted** | 0,72 | [0,68 | 0,76]*** | 1,16 | [1,09 | | 1,22]*** |
| **Deductibles depleted*MHN deployment** |  |  |  |  |  | |  |
| Depleted*Medium-low | 1,11 | [1,03 | 1,20]** | 1,02 | [0,95 | | 1,10] |
| Depleted*Medium-high | 1,16 | [1,08 | 1,25]*** | 1,06 | [0,98 | | 1,15] |
| Depleted*High | 1,18 | [1,09 | 1,27]*** | 1,12 | [1,04 | | 1,21]** |
| **Age in years** | 0,74 | [0,72 | 0,77]*** | 0,73 | [0,71 | | 0,76]*** |
| **Female (yes/no)** | 1,02 | [0,96 | 1,09] | 0,87 | [0,83 | | 0,92]*** |
| **Socioeconomic status score** | 1,04 | [1,01 | 1,08]* | 1,01 | [0,98 | | 1,05] |
| **Number of psychological complaints** | 0,93 | [0,90 | 0,96]*** | 1,13 | [1,10 | | 1,16]*** |
| **Number of psychological disorders** | 0,97 | [0,94 | 1,00]* | 1,26 | [1,23 | | 1,30]*** |
| **Benzodiazepine (yes/no)** | 1,03 | [1,00 | 1,07]* | 1,14 | [1,11 | | 1,18]*** |
| **Antidepressant (yes/no)** | 0,78 | [0,76 | 0,81]*** | 1,22 | [1,19 | | 1,26]*** |

Table 2. Full regression output for being treated in basic mental healthcare of in specialized mental healthcare as a function of mental health nurse deployment, corrected for interaction with depletion of deductibles and confounding by age, gender, socioeconomic status, depletion of deductibles, psychological comorbidities and medication usage.

*P<0.05, **P<0.01, ***P<0.001. MHN= mental health nurse. Socioeconomic status scores were removed from the regression model for specialized mental healthcare, since the model could not converge when socioeconomic status was included, due to the large standard error for socioeconomic statis which made the model more complex without a meaningful reduction of the overall error function.

**Interaction between mental health nurse deployment and depletion of deductibles**


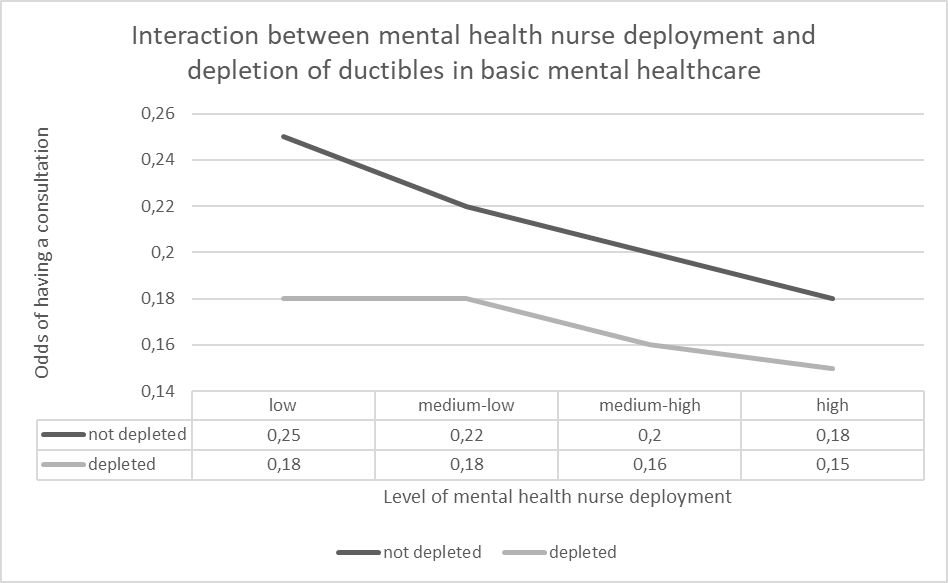

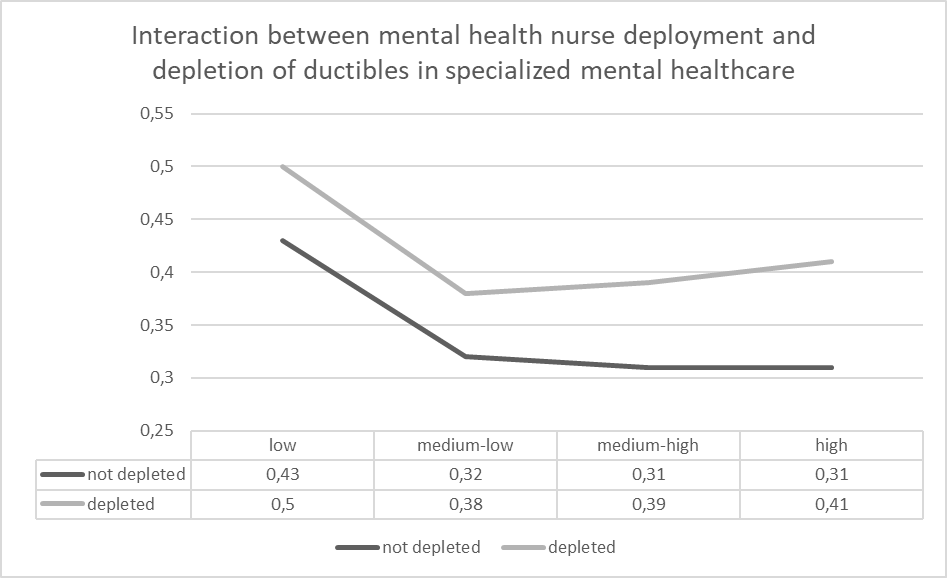

Supplement: Supplementary file 1 — Supplementary Material 1 [file 12875_2024_2402_MOESM1_ESM.docx]
